# Hemophagocytic Lymphohistiocytosis Triggered by Acute Dengue Infection in a Young Adult: A Case Report From the United Arab Emirates

**DOI:** 10.7759/cureus.103135

**Published:** 2026-02-06

**Authors:** Niyas Khalid Ottu Para, Hala Oweis, Seema Rab

**Affiliations:** 1 Internal Medicine, Burjeel Hospital, Abu Dhabi, ARE

**Keywords:** cytokine storm, dengue fever, hemophagocytic lymphohistiocytosis, hyperferritinemia, secondary hlh

## Abstract

Hemophagocytic lymphohistiocytosis (HLH) is a rare, life-threatening hyperinflammatory syndrome characterized by uncontrolled immune activation and cytokine storm. Dengue, a flavivirus transmitted by *Aedes* mosquitoes, is usually self-limiting but may rarely trigger secondary HLH. Persistent fever accompanied by cytopenias, extreme hyperferritinemia, transaminitis, and hypofibrinogenemia should raise suspicion for this diagnosis. Although dengue is not endemic to the United Arab Emirates, a significant outbreak followed unprecedented rainfall and flooding in April 2024, resulting in a surge in imported and locally acquired cases and posing diagnostic challenges for hyperinflammatory conditions such as HLH. We report the case of a 22-year-old male admitted with acute dengue infection complicated by secondary HLH, diagnosed based on extreme hyperferritinemia, bicytopenia, marked transaminitis, and hypofibrinogenemia despite normal triglyceride levels. Early recognition and prompt initiation of corticosteroid therapy led to rapid clinical and biochemical improvement, preventing progression to a full-blown cytokine storm and the need for intensive care admission. This case underscores the importance of maintaining a high index of suspicion for HLH in dengue patients presenting with persistent fever and disproportionate inflammatory markers, particularly in young adults, even in non-endemic regions.

## Introduction

Hemophagocytic lymphohistiocytosis (HLH) is a rare, life-threatening hyperinflammatory syndrome characterized by uncontrolled immune activation resulting from impaired cytotoxic function of natural killer (NK) cells and CD8+ T lymphocytes. This immune dysregulation leads to sustained macrophage activation and excessive release of pro-inflammatory cytokines, including interferon-γ, interleukins (IL-1, IL-2, IL-6, IL-18), and tumor necrosis factor-α, culminating in a cytokine storm, rapid end-organ dysfunction, and high mortality if untreated [1].

HLH is broadly classified into primary (familial) and secondary (acquired) forms. Primary HLH is a pediatric disorder caused by inherited defects in cytotoxic lymphocyte pathways and typically presents in infancy or early childhood. In contrast, secondary HLH occurs in the absence of identifiable genetic mutations and is triggered by infections, malignancies, autoimmune or autoinflammatory disorders, and immunodeficiency states. In adults, secondary HLH predominates, with viral infections, most notably Epstein-Barr virus and dengue virus, representing well-recognized triggers [2,3].

Dengue is a mosquito-borne flaviviral infection caused by four distinct serotypes (DENV-1 to DENV-4) and poses a major global public health burden. Approximately half of the world’s population is at risk, with an estimated 100-400 million infections occurring annually, predominantly in tropical and subtropical regions [4,5]. While dengue is endemic in many parts of Southeast Asia, where dengue-associated HLH has been increasingly reported, it remains uncommon and under-recognized in the Middle East [6]. Diagnostic delays are frequent due to overlapping clinical features with severe dengue, sepsis, and viral hemorrhagic fevers [7]. This distinction is clinically important, as secondary HLH may respond to immunosuppression and treatment of the underlying trigger, without requiring definitive interventions such as hematopoietic stem cell transplantation, typically reserved for primary or malignancy-associated HLH [8].

Clinically, HLH is characterized by persistent fever, cytopenias, liver dysfunction, coagulopathy, and markedly elevated inflammatory markers. Among laboratory parameters, extreme hyperferritinemia is considered a key diagnostic indicator and correlates with disease severity. Although hemophagocytosis on bone marrow or tissue biopsy supports the diagnosis, its absence does not exclude HLH, making biochemical markers, particularly ferritin and soluble interleukin-2 receptor, crucial for early identification [9].

In the United Arab Emirates, HLH remains underreported despite rising dengue incidence, largely due to limited clinical awareness, diagnostic overlap with severe infections, and restricted access to advanced immunological testing. Delayed recognition often results in postponed initiation of immunomodulatory therapy, contributing to increased morbidity and mortality [10]. Early consideration of HLH in dengue patients with unexplained cytopenias and disproportionate hyperferritinemia is therefore essential [11].

We report a case of dengue-triggered secondary HLH in a young adult from the United Arab Emirates, highlighting the critical importance of early recognition and prompt corticosteroid therapy in preventing progression to catastrophic immune-mediated organ failure.

## Case presentation

A 22-year-old previously healthy male presented to the Emergency Department with persistent high-grade fever and early systemic features disproportionate to uncomplicated dengue, including severe myalgia, profound fatigue, generalized erythematous rash, worsening abdominal pain, and persistent headache. At presentation, he appeared acutely unwell and distressed, with systemic symptoms that were disproportionate to the relatively short duration of illness. The initial clinical impression favored an acute viral illness with exanthem, most notably dengue, given the constellation of fever, rash, myalgia, and gastrointestinal symptoms. However, the severity of constitutional symptoms raised early concern for a more aggressive inflammatory process.

The abdominal pain was localized to the epigastric and periumbilical regions, non-radiating, and not associated with vomiting, diarrhea, hematemesis, or melena. The patient reported reduced oral intake and anorexia but denied retro-orbital pain, bleeding manifestations, altered mental status, respiratory symptoms, urinary complaints, recent travel, insect bites, or exposure to sick contacts. He had no prior history of chronic illness, immunosuppression, or regular medication use.

On arrival, the patient was febrile and hypotensive with relative bradycardia, while oxygen saturation was preserved on room air. Although he did not demonstrate overt features of shock, bleeding, altered consciousness, or respiratory distress, he appeared clinically unstable and systemically ill. Physical examination revealed mild abdominal tenderness without guarding or rigidity. There was no hepatosplenomegaly, lymphadenopathy, neck stiffness, or mucocutaneous bleeding.

Initial laboratory investigations (Table 1) demonstrated leukopenia and thrombocytopenia with preserved hemoglobin levels. Serial testing confirmed progressive cytopenias. Liver function tests revealed marked transaminitis, with disproportionately elevated aspartate aminotransferase and alanine aminotransferase levels, accompanied by elevated gamma-glutamyl transferase. Notably, total and direct bilirubin levels remained within normal limits throughout the illness.

**Table 1:** Serial laboratory parameters demonstrating lab abnormalities consistent with secondary hemophagocytic lymphohistiocytosis in dengue infection. Summary of hematological, biochemical, inflammatory, coagulation, lipid, and infectious laboratory investigations with reference ranges. ANA, antinuclear antibody; Anti-CCP, anti-cyclic citrullinated peptide antibody; ALT, alanine aminotransferase; AST, aspartate aminotransferase; CMV, cytomegalovirus; CRP, C-reactive protein; dsDNA, double-stranded deoxyribonucleic acid; EBV, Epstein-Barr virus; GGT, gamma-glutamyl transferase; HDL, high-density lipoprotein; HHV-6, human herpesvirus 6; IgG, immunoglobulin G; IgM, immunoglobulin M; INR, international normalized ratio; LDL, low-density lipoprotein; LDH, lactate dehydrogenase; NS1, non-structural protein 1; PCR, polymerase chain reaction

Category	Parameter	Result	Reference range/cut-off
Hematology	Hemoglobin	14.2 g/dL	13.0-17.0 g/dL
	White blood cell count	2.06 × 10⁹/L	4.0-11.0 × 10⁹/L
	Platelet count (lowest)	103 × 10⁹/L	150-450 × 10⁹/L
	Platelet count (later)	321 × 10⁹/L	150-450 × 10⁹/L
Liver enzymes and function	AST (peak)	846 U/L	5-40 U/L
	AST (subsequent)	129 U/L	5-40 U/L
	ALT (peak)	678 U/L	5-41 U/L
	ALT (subsequent)	522 U/L	5-41 U/L
	GGT	139 U/L	10-71 U/L
	Total bilirubin	7.8 µmol/L	5-21 µmol/L
	Direct bilirubin	4.7 µmol/L	0-5 µmol/L
Inflammatory and cell turnover	Ferritin (peak)	13,355 µg/L	30-400 µg/L
	Ferritin (subsequent)	9,494 µg/L	30-400 µg/L
	Ferritin (later)	1,720 µg/L	30-400 µg/L
	LDH	605 U/L	135-225 U/L
	C-reactive protein (CRP)	1.1 mg/L	<5 mg/L
	Procalcitonin	0.05 ng/mL	<0.5 ng/mL
	Lactic acid	1.1 mmol/L	0.5-2.2 mmol/L
Coagulation	Fibrinogen	1.5 g/L	2.0-4.0 g/L
	INR	1.2	0.9-1.2
Lipid profile	Total cholesterol	2.61 mmol/L	<5.2 mmol/L
	Triglycerides	1.4 mmol/L	<1.7 mmol/L
	HDL cholesterol	0.52 mmol/L	>1.0 mmol/L
	LDL cholesterol (calculated)	2.09 mmol/L	<3.0 mmol/L
	Cholesterol/HDL ratio	4.98	<4.5
Protein electrophoresis	Total protein	64.3 g/L	66-87 g/L
	Albumin	4.0 g/dL	3.5-5.0 g/dL
	Alpha-1 globulin	0.40 g/dL	0.1-0.3 g/dL
	Alpha-2 globulin	0.80 g/dL	0.6-1.0 g/dL
	Beta-1 globulin	0.40 g/dL	0.4-0.6 g/dL
	Beta-2 globulin	0.20 g/dL	0.2-0.5 g/dL
	Gamma globulin	0.90 g/dL	0.7-1.6 g/dL
Autoimmune/rheumatology	ANA (IFT)	Negative	Negative
	ANA titer	1:100	<1:160
	Anti-dsDNA	10 IU/mL	<30 IU/mL
	Rheumatoid factor	0.88 IU/mL	<14 IU/mL
	Anti-CCP	8 U/mL	<20 U/mL
Infection - dengue	Dengue NS1 antigen	Positive	Negative
	Dengue RT-PCR	Positive	Negative
	Dengue IgM	Positive	Negative
	Dengue IgG	Negative	Negative
Other infections	Chikungunya IgM	Negative	Negative
	Chikungunya IgG	Negative	Negative
	EBV quantitative PCR	Not detected	Not detected
	HHV-6 PCR	Not detected	Not detected
	Parvovirus B19 PCR	Not detected	Not detected
	CMV IgM	Non-reactive	Non-reactive
	CMV IgG	Non-reactive	Non-reactive
	Hepatitis B surface antigen	Non-reactive	Non-reactive
	Hepatitis C antibody	Non-reactive	Non-reactive
	Hepatitis A total antibody	Non-reactive	Non-reactive
	Leptospira IgM	Negative	Negative
	Leptospira IgG	Negative	Negative
	Borrelia burgdorferi IgM	Negative	Negative
	Borrelia burgdorferi IgG	Negative	Negative
	Malaria antibody	Negative	Negative
	Influenza A antigen	Negative	Negative
	Influenza B antigen	Negative	Negative
	Throat culture	No growth	No growth

A striking discordance soon became apparent between the patient’s evolving biochemical profile and the expected laboratory pattern of uncomplicated dengue infection. Markers of inflammation and cellular turnover revealed extreme hyperferritinemia and elevated lactate dehydrogenase levels, findings grossly disproportionate to the clinical severity typically observed in routine viral infections. In contrast, inflammatory markers commonly associated with bacterial infection, including C-reactive protein (CRP) and procalcitonin, remained within normal limits, and serum lactate levels were normal, arguing against septic shock or tissue hypoperfusion.

Coagulation studies revealed hypofibrinogenemia with preserved international normalized ratio. Lipid profile analysis demonstrated reduced high-density lipoprotein cholesterol levels, while triglyceride and low-density lipoprotein levels remained within normal ranges. Serum protein electrophoresis showed mild hypoproteinemia with a reactive acute-phase pattern, characterized by a mild increase in alpha-1 globulin fraction and borderline reduction in beta-2 globulin fraction, with preserved albumin and gamma globulin levels. There was no evidence of monoclonal gammopathy.

Autoimmune screening, including antinuclear antibodies, anti-double-stranded DNA antibodies, rheumatoid factor, and anti-cyclic citrullinated peptide antibodies, was negative. Infectious investigations confirmed acute dengue infection, evidenced by positive dengue NS1 antigen and IgM antibodies in the absence of IgG antibodies, consistent with primary dengue infection. Dengue RT-PCR testing was also positive. Extensive evaluation for alternative infectious etiologies, including chikungunya, Epstein-Barr virus, cytomegalovirus, parvovirus B19, hepatitis viruses, leptospirosis, malaria, borrelia, influenza, and bacterial pathogens, was uniformly negative.

At this juncture, the combination of persistent high-grade fever, progressive cytopenias, severe transaminitis, hypofibrinogenemia, and extreme hyperferritinemia-occurring in the absence of bacterial inflammatory response-raised strong suspicion for an evolving hyperinflammatory syndrome. Clinicians recognized that the patient was approaching a critical inflection point, with laboratory evidence suggestive of an impending cytokine storm rather than isolated severe dengue infection. Secondary HLH triggered by acute dengue infection was therefore strongly suspected.

**Figure 1:**
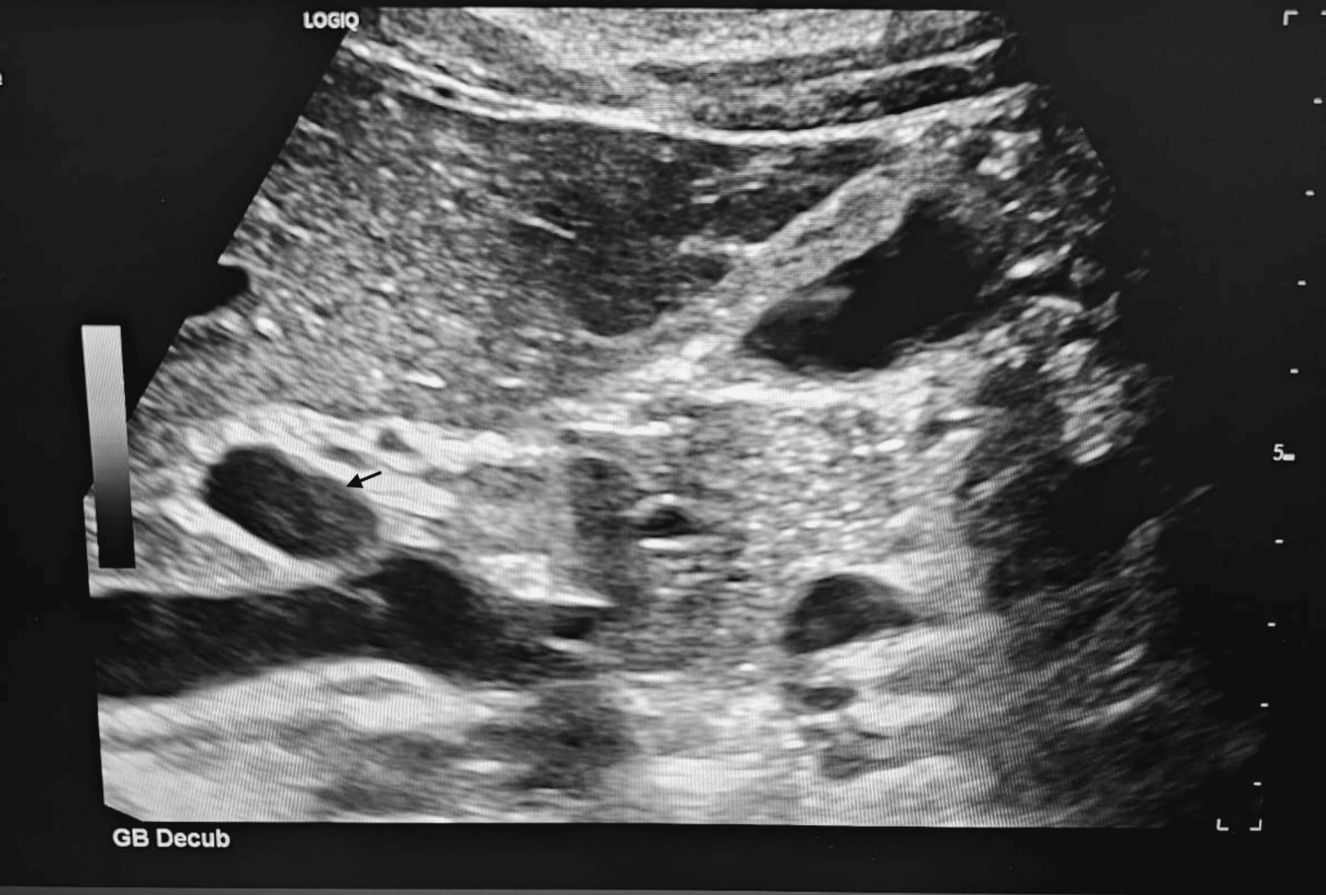
Abdominal ultrasound showing gallbladder wall edema with minimal intraperitoneal free fluid. Abdominal ultrasound image depicting minimal free intraperitoneal fluid and diffuse gallbladder wall thickening with edema. Small gallbladder polyps measuring up to 2.8 mm were also visualized. The liver and spleen appeared normal in size.

**Figure 2:**
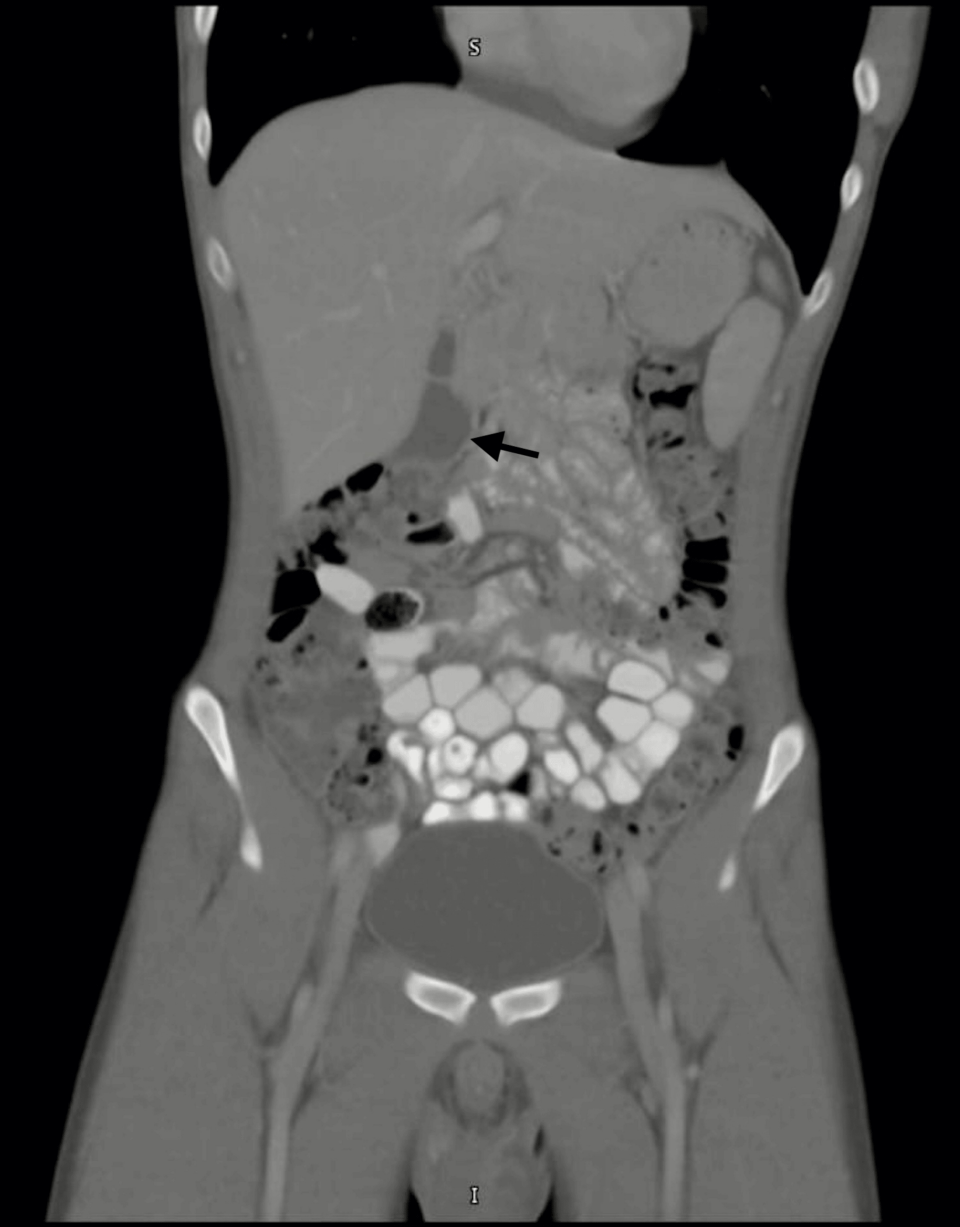
Contrast-enhanced CT abdomen demonstrating gallbladder wall enhancement with minimal hepatorenal free fluid. Contrast-enhanced computed tomography of the abdomen and pelvis showing mild reactive enhancement of the gallbladder wall and minimal free fluid in the hepatorenal fossa. No focal hepatic or splenic lesions were identified. These findings are supportive of inflammatory changes associated with acute dengue infection.

Imaging studies were performed to evaluate for intra-abdominal pathology and organomegaly. Abdominal ultrasonography (Figure 1) demonstrated mild free intraperitoneal fluid and a thickened, edematous gallbladder wall with a few small gallbladder polyps measuring up to 2.8 mm, without evidence of hepatomegaly or splenomegaly. Contrast-enhanced computed tomography (Figure 2) of the abdomen and pelvis revealed mild reactive gallbladder wall enhancement and minimal fluid in the hepatorenal fossa, without focal hepatic or splenic lesions. Plain abdominal radiography demonstrated a few air-fluid levels within bowel loops, consistent with enteritis.

Early recognition of this evolving hyperinflammatory state allowed prompt initiation of immunomodulatory therapy, resulting in rapid clinical and biochemical improvement and preventing progression to fulminant cytokine storm, multiorgan failure, and need for intensive care admission.

Clinical course

Based on the initial clinical presentation and early laboratory findings, the patient was initially managed as a case of acute dengue fever (Table 2). However, during hospitalization, he developed progressive thrombocytopenia, worsening transaminitis, hypofibrinogenemia, and extreme hyperferritinemia, prompting concern for an evolving hyperinflammatory syndrome. The persistence of high-grade fever in association with cytopenias involving multiple cell lines, markedly elevated ferritin levels, and rising lactate dehydrogenase raised a strong suspicion for dengue-triggered secondary HLH, fulfilling multiple HLH-2004 diagnostic criteria.

**Table 2:** Timeline of clinical course, key investigations, management, and response to treatment. This table outlines the day-wise progression of clinical features, laboratory and imaging findings, therapeutic interventions, and corresponding clinical response during hospitalization. The trend demonstrates the evolution of hyperinflammatory features consistent with secondary hemophagocytic lymphohistiocytosis triggered by dengue infection and the subsequent improvement following initiation of corticosteroid therapy. ALT, alanine aminotransferase; AST, aspartate aminotransferase; LDL, low-density lipoprotein; PCR, polymerase chain reaction

Hospital day	Clinical features	Key laboratory/imaging findings	Management	Clinical response
Day 0	High-grade fever, generalized erythematous rash, abdominal pain, hypotension, distress	Leukopenia, thrombocytopenia, elevated transaminases	IV fluids, empiric antibiotics, close monitoring	Persistent fever
Day 1	Ongoing fever, rash, GI symptoms	Ferritin 13,355 ng/mL; AST 846 U/L; ALT 569 U/L; fibrinogen 1.5 g/L	Expanded infectious and inflammatory work-up	Hyperinflammatory state suspected
Day 2	Hemodynamic instability improving	Dengue PCR positive; cytopenias persist	Dexamethasone initiated	Fever curve improves
Day 3	Rash fading, afebrile	Ferritin ↓ to 9,494 ng/mL; LDH ↓	Steroids continued	Marked biochemical improvement
Day 4	Clinically stable	Ferritin ↓ to 7,146 ng/mL; platelets rising	Steroid taper planning	Recovery phase
Day 5	Asymptomatic	Normalizing blood counts and liver enzymes	Discharged on oral steroid taper	Sustained recovery

Empiric intravenous broad-spectrum antibiotic therapy with ceftriaxone, along with oral doxycycline, was initiated early in the hospital course to provide coverage for potential bacterial co-infection while awaiting microbiological results. In parallel, systemic corticosteroid therapy was commenced due to high clinical suspicion for secondary HLH. Serial laboratory monitoring demonstrated that serum ferritin levels, which had peaked above 13,000 µg/L early in the hospital course, began to decline steadily following initiation of immunomodulatory therapy. Platelet counts, which had reached a nadir of 103 × 10⁹/L, showed progressive recovery over subsequent days after steroid initiation.

Further diagnostic evaluation confirmed acute dengue infection, with dengue NS1 antigen positivity early in the course and dengue IgM positivity confirmed later during hospitalization, consistent with primary dengue infection. Extensive microbiological and immunological investigations, including blood cultures, malaria smear, viral hepatitis panel, Epstein-Barr virus polymerase chain reaction, cytomegalovirus serology, chikungunya testing, and autoimmune screening, were all negative, supporting dengue as the primary trigger for the hyperinflammatory state. Radiological imaging, including abdominal ultrasonography and contrast-enhanced computed tomography, demonstrated mild ascites and reactive gallbladder wall thickening without evidence of hepatosplenomegaly or focal hepatic or splenic lesions.

Clinically, the patient demonstrated steady improvement with resolution of fever, stabilization of hemodynamic parameters, and progressive normalization of laboratory abnormalities. He did not develop organ failure and did not require intensive care admission. The patient was discharged after six days of hospitalization in a stable condition with improving biochemical markers and was prescribed a tapering course of oral corticosteroids, with close outpatient follow-up arranged.

Diagnostic assessment

Based on the HLH-2004 diagnostic criteria, the patient fulfilled ≥4 criteria (Table 3).

**Table 3:** Application of HLH-2004 diagnostic criteria in this case. Diagnostic criteria adapted from Henter et al. [12]. HLH, hemophagocytic lymphohistiocytosis; NK, natural killer

HLH-2004 criterion	Patient findings	Criterion met
Fever	Persistent high-grade fever	Yes
Cytopenias (≥2 lineages)	Leukopenia + thrombocytopenia	Yes
Hyperferritinemia	Ferritin 13,355 ng/mL	Yes
Hypofibrinogenemia	Fibrinogen 1.5 g/L	Yes
Splenomegaly	Absent on imaging	No
Hemophagocytosis	Not assessed	-
Elevated soluble CD25	Not available	-
Decreased NK cell activity	Not available	-

Based on the HLH-2004 diagnostic criteria, as originally described by Henter et al. [12], the patient met four criteria: persistent fever, bicytopenia, hyperferritinemia, and hypofibrinogenemia, with additional supportive features including markedly elevated liver enzymes. Although the patient fulfilled four of the eight HLH-2004 diagnostic criteria, it is well recognized that these criteria were originally developed for pediatric familial HLH and may lack sensitivity in adult infection-associated HLH. In adult patients, particularly those with infection-triggered disease, secondary HLH may be diagnosed even when fewer than five HLH-2004 criteria are met, and application of the H-score, a validated diagnostic tool for reactive HLH in adults, further supported a high probability of secondary HLH in this case. An H-score calculation indicated a high probability (>80%) of secondary HLH. Bone marrow biopsy and soluble IL-2 receptor testing were deferred due to strong clinical and biochemical evidence and the patient’s improving condition.

The final diagnosis was secondary HLH triggered by acute dengue fever.

## Discussion

This case describes a 22-year-old male patient in the United Arab Emirates who developed secondary HLH triggered by acute dengue infection, presenting with persistent high-grade fever, cytopenias, hypofibrinogenemia, and extreme hyperferritinemia, fulfilling multiple HLH-2004 diagnostic criteria and demonstrating organ dysfunction disproportionate to classical dengue severity [13]. Although the HLH-2004 diagnostic criteria require fulfillment of five out of eight criteria, these criteria were originally developed for pediatric familial HLH and are known to have reduced sensitivity in adult, infection-associated HLH. In adult patients, particularly those with infection-triggered disease, secondary HLH may be diagnosed even when fewer criteria are met. In this context, the H-score has been validated as a diagnostic tool for reactive HLH in adults and provides a probabilistic assessment of disease likelihood. In our patient, the calculated H-score indicated a high probability (>80%) of secondary HLH, supporting the diagnosis despite fulfillment of four HLH-2004 criteria [14]. The presence of normal CRP and procalcitonin levels despite severe clinical and biochemical derangement provided an important clue distinguishing HLH from bacterial sepsis. Importantly, a normal CRP does not exclude HLH, whereas extreme hyperferritinemia remains a key early diagnostic biomarker. Early recognition in this patient allowed the timely initiation of corticosteroid therapy, preventing progression to a full-blown cytokine storm, multiorgan failure, and the need for intensive care admission. Similar reports suggest that early steroid therapy alone may be sufficient in infection-triggered HLH when initiated promptly [15].

The patient’s condition improved following early corticosteroid therapy combined with supportive care, underscoring the importance of prompt recognition and targeted immunosuppression. The cornerstone of HLH management involves immunosuppression, most commonly corticosteroids, alongside treatment of the underlying trigger. In this case, corticosteroid therapy resulted in rapid clinical stabilization and progressive biochemical improvement without the need for cytotoxic agents or advanced immunomodulatory therapies.

Case reports from tropical and subtropical regions, particularly Southeast Asia and the Americas, describe similar presentations, often involving young adults with dengue complicated by prolonged fever beyond the expected critical phase, cytopenias, coagulopathy, and evidence of hemophagocytosis on bone marrow examination [16]. In the present case, the absence of underlying malignancy, autoimmune disease, or genetic predisposition strongly supports dengue infection as the sole triggering factor. Dengue-associated HLH has been reported in a subset of severe dengue cases, particularly during secondary infections. A retrospective study from Singapore demonstrated markedly elevated ferritin levels (mean 34,740 ng/mL) and favorable outcomes with short courses of corticosteroids, closely mirroring our patient’s course, in whom ferritin exceeded 13,000 ng/mL and declined rapidly following steroid initiation [17]. This contrasts with malignancy-associated HLH, where aggressive regimens such as HLH-94 protocols incorporating etoposide are often required. In infection-triggered HLH, including dengue-associated cases, less intensive immunosuppression may be sufficient and avoids the risks associated with profound cytotoxic therapy [18].

The implications of this case are particularly relevant in the UAE, where dengue incidence has increased following unprecedented flooding and heavy rainfall in 2024, creating favorable breeding conditions for *Aedes* mosquitoes. This surge reflects a broader global trend, with more than 14 million dengue cases reported worldwide in 2024, the highest number on record, and continued increases into 2025. Between January and July 2025 alone, over four million cases and more than 3,000 deaths were reported to the World Health Organization from 97 countries [4]. In the UAE, local dengue transmission has been documented since 2023, driven by climate variability, unseasonal rainfall, and rising temperatures associated with climate change. Unlike endemic tropical regions where dengue-associated HLH follows predictable seasonal patterns, outbreaks in the Middle East represent an emerging and under-recognized clinical challenge, underscoring the intersection between climate-driven vector-borne diseases and hyperinflammatory syndromes in non-traditional settings.

From a clinical perspective, this case emphasizes the need for early consideration of HLH in dengue patients with unrelenting fever, progressive cytopenias, or disproportionately elevated inflammatory markers, using tools such as the H-score where available. Public health strategies should prioritize mosquito control, surveillance, and clinician awareness to mitigate outbreaks in vulnerable populations, in line with recommendations from the UAE Ministry of Health [19]. Early identification is particularly important given that mortality rates in untreated dengue-associated HLH have been reported to range from 4.5% to over 40%, whereas timely intervention substantially improves outcomes [1]. In this patient, early recognition of bicytopenia and coagulopathy, evidenced by hypofibrinogenemia, mirrored established dengue-HLH patterns, where mortality can reach 14-20% without prompt treatment [20].

Management in this case included empiric antimicrobial coverage with ceftriaxone and doxycycline to address potential bacterial co-infection, alongside corticosteroid therapy. The patient achieved complete clinical and biochemical recovery without requiring additional immunomodulators such as etoposide or biologic agents, highlighting the effectiveness of early, conservative immunosuppression in infection-triggered HLH. This approach aligns with emerging literature favoring steroid-based regimens in selected cases, minimizing the risk of secondary infections while achieving disease resolution [2].

Several limitations warrant consideration. As a single-patient case report, generalizability is limited, and genetic testing for primary HLH predisposition was not performed. Bone marrow biopsy was also deferred; however, the diagnosis was strongly supported by the patient’s clinical presentation and fulfillment of HLH-2004 criteria, including persistent fever, cytopenias, extreme hyperferritinemia, hypofibrinogenemia, and hepatic dysfunction. Additionally, underreporting of dengue cases in the region may obscure the true incidence of dengue-associated HLH in the UAE.

This case highlights the urgent need for targeted public health responses in the UAE, including enhanced mosquito surveillance, climate-resilient vector control strategies, and integration of hyperinflammatory syndromes into dengue management pathways. As global dengue incidence continues to rise, proactive clinical vigilance and early intervention may prevent atypical and potentially catastrophic immune-mediated complications in emerging non-endemic regions. Future research should focus on longitudinal studies in newly affected regions, biomarker-guided diagnostic strategies, and comparative trials evaluating steroid-only versus combination regimens to optimize outcomes while avoiding overtreatment.

Outcome and follow-up

The patient demonstrated rapid clinical and biochemical improvement within 72 hours of corticosteroid initiation. Serial laboratory monitoring showed a marked decline in serum ferritin levels, normalization of platelet counts, and gradual improvement in liver enzyme abnormalities. He remained hemodynamically stable throughout hospitalization and did not require intensive care admission, intravenous immunoglobulin therapy, or cytotoxic agents such as etoposide. The patient was discharged in stable condition on a tapering course of oral corticosteroids, with outpatient follow-up arranged to ensure continued clinical recovery and laboratory normalization.

Learning points

In patients with dengue, ferritin levels exceeding 10,000 µg/L should raise a strong suspicion for secondary HLH, even when traditional markers such as CRP remain within normal limits. The development of worsening cytopenias and liver dysfunction in the course of illness should further prompt consideration of this hyperinflammatory complication. Early recognition is crucial, as timely initiation of corticosteroid therapy can be effective in controlling infection-triggered HLH and preventing clinical deterioration. This awareness is especially important in non-endemic regions where rising dengue incidence may lead to delayed recognition of this rare but life-threatening condition.

## Conclusions

Secondary HLH is a rare but potentially fatal complication of dengue fever, resulting from uncontrolled hyperinflammatory immune activation. Extreme hyperferritinemia in the setting of dengue infection should prompt urgent evaluation for HLH, particularly when accompanied by unexplained cytopenias and organ dysfunction disproportionate to the severity of dengue alone. This case underscores the critical importance of early recognition and timely initiation of corticosteroid therapy, which can be life-saving and may prevent progression to cytokine storm, multiorgan failure, or the need for aggressive immunosuppressive regimens.

This report adds to the limited regional literature on dengue-associated HLH in the Middle East, a region experiencing increasing dengue incidence driven by climate variability, urbanization, and expanding mosquito habitats. Diagnostic delays remain common due to overlapping clinical features with severe dengue and sepsis, highlighting the need for heightened clinical vigilance and structured diagnostic pathways. Early recognition, systematic exclusion of mimics, and coordinated care involving laboratory, microbiology, radiology, and specialty services where appropriate may help inform tailored diagnostic and management approaches for young adults in the United Arab Emirates and other emerging dengue-affected regions.
